# Use of risk scores to identify lower and higher risk subsets among COMPASS‐eligible patients with chronic coronary syndromes. Insights from the CLARIFY registry

**DOI:** 10.1002/clc.23505

**Published:** 2020-12-04

**Authors:** Arthur Darmon, Gregory Ducrocq, Adam Jasilek, Laurent Feldman, Emmanuel Sorbets, Roberto Ferrari, Ian Ford, Jean‐Claude Tardif, Michal Tendera, Kim M. Fox, Philippe Gabriel Steg

**Affiliations:** ^1^ Université de Paris, Assistance Publique–Hôpitaux de Paris Paris France; ^2^ FACT, French Alliance for Cardiovascular Trials, Hôpital Bichat Paris France; ^3^ Robertson Centre for Biostatistics University of Glasgow Glasgow UK; ^4^ Assistance Publique ‐ Hôpitaux de Paris, Hôtel Dieu Centre de Diagnostic et de Thérapeutique Paris France; ^5^ INSERM U‐1148, Laboratory for Vascular Translational Science Paris France; ^6^ Department of Cardiology University Hospital of Ferrara and Maria Cecilia Hospital, GVM Care and Research Cotignola Italy; ^7^ Montreal Heart Institute Université de Montréal Montreal Canada; ^8^ Department of Cardiology and Structural Heart Disease Medical University of Silesia, School of Medicine in Katowice Katowice Poland; ^9^ National Heart and Lung Institute, Royal Brompton Hospital Imperial College London UK

**Keywords:** bleeding risk, chronic coronary syndromes, COMPASS, ischemic risk, risk score, rivaroxaban

## Abstract

**Background:**

The COMPASS trial showed a reduction of ischemic events with low‐dose rivaroxaban and aspirin in chronic coronary syndromes (CCS) compared with aspirin alone, at the expense of increased bleeding.

**Hypothesis:**

The CHA_2_DS_2_VaSc Score, REACH Recurrent Ischemic (RIS), and REACH Bleeding Risk Score (BRS) could identify patients with a favorable trade‐off between ischemic and bleeding events, among COMPASS‐eligible patients.

**Methods:**

We identified the COMPASS‐eligible population within the CLARIFY registry (>30.000 patients with CCS). High‐bleeding risk patients (REACH BRS > 10) were excluded, as in the COMPASS trial. Patients were categorized as low (0–1) or high (≥ 2) CHA_2_DS_2_VaSc; low (0–12) or intermediate (13–19) REACH RIS, and low (0–6) or intermediate (7–10) REACH BRS. Ischemic outcome was the composite of cardiovascular death, myocardial infarction or stroke. Bleeding was defined as serious bleeding (haemorrhagic stroke, hospitalization for bleeding, transfusion).

**Results:**

The COMPASS‐eligible population comprised 5.142 patients with ischemic and bleeding outcome of 2.3 (2.1–2.5) and 0.5 (0.4–0.6) per 100 patient‐years, respectively. Patients with intermediate REACH RIS (n = 1934 [37.6%]) had the higher ischemic risk (3.0 [2.6–3.4]) with similar bleeding risk (0.5 [0.4–0.7]) as the overall population. Patients with low CHA_2_DS_2_VaSc (n = 229 [4.4%]) had a very low ischemic risk (0.6 [0.3–1.3]) with similar bleeding risk (0.5 [0.2–1.1]).

**Conclusions:**

Intermediate REACH RIS identified potential optimal candidates for adjunction of low‐dose rivaroxaban while patients with low CHA_2_DS_2_VaSc score .appears unlikely to benefit from the COMPASS regimen. None of the three risk scores predicted the occurrence of serious bleeding.

## 1 INTRODUCTION

In patients with chronic coronary syndromes (CCS) or peripheral arterial disease (PAD), antithrombotic treatment represents a cornerstone of medical therapy. This has been for a long time limited to single antiplatelet therapy alone. However, there remains strong evidence of a high residual thrombotic risk in both CCS and PAD patients.^1^, ^2^, ^3^, ^4^ In that sense, several antithrombotic regimens have been evaluated in order to reduce the occurrence of recurrent ischemic events, based on potent antiplatelet therapy, or on the combination of antiplatelet therapy with oral anticoagulant therapy.^5^, ^6^, ^7^ The COMPASS^6^ trial demonstrated the efficacy of a combination of low‐dose rivaroxaban and aspirin in reducing ischemic events in a broad population of high‐risk patients with CCS and/or PAD, but with a 70% relative increase in the risk of major bleeding. Recently, the ESC guidelines^8^ recommended the COMPASS regimen in patients at high ischemic risk without high bleeding risk. However, the identification of such patients in routine clinical practice can be challenging, particularly as some clinical or biological conditions correlate to both ischemic and bleeding risk. For quantitative assessment of both ischemic and bleeding risk, risk scores have proven to be useful in various clinical settings, such as atrial fibrillation.

In the present analysis, we used data from the CLARIFY Registry, a large international registry of more than 30 000 patients with CCS, to evaluate the performance of established ischemic and bleeding risk scores: the CHA_2_DS_2_VaSc score, the REACH Recurrent Ischemic Score (RIS) and the REACH Bleeding Risk Score (BRS) in order to attempt to identify patients with the most favorable trade‐off between ischemic and bleeding events, among CCS patients eligible to COMPASS.

## 2 METHODS

### 2.1 The CLARIFY registry

CLARIFY^9^, ^10^, ^11^ was an international, prospective, longitudinal registry of outpatients with CCS, and enrolled 31 593 patients from November 2009 until July 2010 in 45 countries. Participation in the registry did not interfere with clinical management and was strictly observational. Patients were followed yearly for up to 5 years. An ECG was recorded at each visit. The rationale, design, and long‐term outcomes of CLARIFY registry participants have been described in detail.^9^, ^10^, ^12^, ^13^

To be eligible for enrolment, patients had to have any of the following: documented myocardial infarction or history of myocardial revascularization—either by percutaneous coronary intervention (PCI) or coronary artery bypass grafting (CABG) more than 3 months earlier—or documented coronary artery disease with coronary angiography showing at least one coronary stenosis of more than 50%, or chest pain with proven myocardial ischemia (using stress ECG, stress echocardiography, or myocardial imaging). Patients were excluded if they had been hospitalized for CV disease within the last 3 months, had a planned revascularization, or any condition hampering the participation or the 5‐year follow‐up, including advanced heart failure. The study was conducted in accordance with the Declaration of Helsinki, and local ethical approval was obtained before recruitment. All patients gave written informed consent.

### 2.2 Identification of the COMPASS‐eligible population within the CLARIFY registry

In order to identify the subset of patients eligible to COMPASS within the CLARIFY registry, we proceeded as follows. First we applied the main COMPASS exclusion criteria: patients with high bleeding risk (based on the calculation of a REACH BRS > 10), severe renal failure with estimated glomerular filtration rate (eGFR) less than 15 ml/min, dual antiplatelet therapy (DAPT) or on oral anticoagulant, severe heart failure (i.e., NYHA III and/or left ventricular ejection fraction <30%), or history of haemorrhagic stroke were excluded. Second, we applied the COMPASS inclusion criteria to the remaining population: (1) PAD patients—encompassing peripheral artery disease and/or carotid disease—were included regardless of age; (2) CCS patients older than 65 years; and (3) CCS patients younger than 65 years had to fulfill the COMPASS 'enrichment criteria' (i.e., documented atherosclerosis or revascularization involving at least two vascular beds) or at least two additional risk factors (i.e., current smoker, diabetes, history of ischemic stroke more than 1 month ago, moderate renal failure with eGFR <60 ml/min, and heart failure with NYHA Class 1 or 2). CCS patients <65 years, without any enrichment criteria were considered ineligible to COMPASS. Thus, patients without exclusion criteria, and fulfilling the COMPASS inclusion criteria constituted the COMPASS‐eligible subset within the CLARIFY registry.

### 2.3 Definition of risk scores

#### 2.3.1 CHA_2_DS_2_VaSc Score

The CHA_2_DS_2_VaSc (cardiac failure, hypertension, Age > 75 [Doubled], diabetes, stroke [Doubled] vascular disease, age 65–74 and sex category [female]) Score have been validated for estimating the risk of thromboembolic stroke in atrial fibrillation (AF) patients.^14^, ^15^ According to its validated use in AF, patients with CHA_2_DS_2_VaSc score of 0–1 were categorized in low‐intermediate risk, and patients with ≥2 as high risk.

#### 2.3.2 The REACH Recurrent Ischemic Score

The REACH RIS,^16^ developed from the REACH registry, establishes a score for the prediction of recurrent ischemic events, in the REACH population of patients with chronic vascular disease. Items used are sex, age, smoking status, diabetes mellitus, body mass index, number of vascular beds with atherothrombosis disease, cardiovascular event in past year, congestive heart failure, atrial fibrillation, statin therapy, aspirin therapy, and country of residence. This score ranges from 0 (< 1% of risk of recurrent event in the next 20 months) to 29 (> 50% of risk) and predicts recurrent cardiovascular (CV) events.

Patients with REACH RIS between 0 (< 1% of risk) and 12 (5.4% of risk) were classified as low risk, while patients with score ranging from 13 (6.3% of risk) to 19 (15.0% of risk) were in the intermediate group. Patients with REACH RIS score ≥ 20 were categorized in the high‐risk group (> 17% of risk over 20 months).

#### 2.3.3 The REACH Bleeding Risk Score

The REACH BRS^17^ has been developed and validated for prediction of serious bleeding (defined as nonfatal haemorrhagic stroke or bleeding leading to both hospitalization and transfusion) in outpatients with chronic vascular disease in the REACH registry.^18^ The following variables are included for the calculation of the score, which ranges from 0 to 23: age, peripheral artery disease, chronic heart failure, diabetes mellitus, hypercholesterolemia, hypertension, smoking, antiplatelet agents, and oral anticoagulants are assigned a number of points.

Patients with score ranging from 0 to 6 were classified as low REACH BRS (0.46% of risk over a two‐year period), patients with score ranging between 7 and 10 as intermediate (approximately 1.1% of risk over a 2 year period) and patients with >10 as high bleeding risk (> 2.76% of risk). As a reminder, patients with high bleeding risk defined by a REACH BRS > 10 were excluded, according to COMPASS exclusion criteria.

### 2.4 Outcomes definition

For the present analysis, the ischemic outcome was a composite of CV death, myocardial infarction (MI) or stroke. The bleeding outcome was “serious bleeding” a composite of bleeding leading to either admission to hospital and/or transfusion, or haemorrhagic stroke. Both outcomes are expressed per 100 patients year.

### 2.5 Statistical analysis

Baseline characteristics are expressed as mean ± SD for continuous variables and categorical variables as frequency and percentage. Baseline characteristics and outcomes were compared between subgroups according to the distribution of the risk score, to allow statistical comparison between mutually exclusive subgroups. Continuous and categorical baseline variables were compared using analysis of variance and chi‐square tests, respectively. The incidence rates were obtained for the specified sub‐population, from an unadjusted generalized linear models with a Poisson distribution, and a Log link, accounting for each patient's follow‐up time, in years, available by including the log of their follow‐up time as an offset in the model. A *p*‐value of <.05 was considered statistically significant. We used the SAS software (version 9.4) for the analyses.

## 3 RESULTS

### 3.1 Baseline characteristics of the CLARIFY‐COMPASS‐eligible population

Among the 32 073 patients included in the CLARIFY registry, 17 518 patients had missing data regarding inclusion and/or exclusion criteria precluding precise evaluation. Therefore, 15 185 patients were fully evaluable regarding eligibility for the COMPASS trial. Of these, 6540 had at least one exclusion criteria (43.1%), and 3503 patients (23.1%) were CCS patients aged <65 years old without any of the enrichment criteria required for eligibility. The COMPASS‐eligible population thus comprised 5142 patients (33.9%).

Baseline characteristics of the study population are shown in Table 1. Mean age was 68.1 ± 7.9 years and 73.5% of the patients were male. Approximately half of the population had a history of myocardial infarction (n = 2905, 56.5%) and PCI (n = 2464, 47.9%), 32.4% (n = 1667) had previous CABG, 7.9% (n = 404) had a history of stroke and 17.7% (n = 912) had a history of congestive heart failure. Major cardiovascular risk factors were highly prevalent with 76.7% (n = 3945) having hypertension, 34.1% (n = 1753) diabetes, 84.3% (n = 4334) dyslipidaemia and 53.1% (n = 2733) who were active or former smokers.

**TABLE 1:** Baseline characteristics of the overall CLARIFY‐COMPASS‐Eligible population, and according to the CHA_2_DS_2_VaSc score, the REACH bleeding risk score and the REACH recurrent ischemic score

	CLARIFY‐COMPASS‐eligible n = 5.142	Low‐intermediate CHA_2_DS_2_VaSc (0–1) n = 229	High CHA_2_DS_2_VaSc (≥2) n = 4913	*p*	Low REACH Bleeding Risk Score N = 833	Intermediate REACH Bleeding Risk Score n = 4309	*p*	Low REACH Recurrent Ischemic Score n = 3184	Intermediate REACH Recurrent Ischemic Score n = 1934	*p*
Age (years ± SD)	68.1 ± 7.9	64.5 ± 7	68.2 ± 7.9	<.0001	61.8 (8.1)	69.3 (7.3)	<.0001	67.9 ± 7.5	68.3 ± 8.5	.22
Age > 75 years, n (%)	788 (15.3%)	0	788 (16.0%)	<.0001	5 (0.6%)	783 (18.2%)	<.0001	408 (12.8%)	376 (19.4%)	<.0001
BMI, mean ± SD	28.1 ± 4.5	27.1 ± 4.0	28.1 ± 4.5	<.0001	27.9 ± 4.4	28.1 ± 4.5	.22	28 ± 4.3	28.3 ± 4.7	.006
Male, n (%)	3.780 (73.5%)	227 (99.1%)	3553 (72.3%)	<.0001	635 (76.2%)	3145 (73.0%)	.05	2219 (69.7%)	1542 (79.7%)	<.0001
Medical history										
Myocardial infarction, n (%)	2.905 (56.5%)	31 (13.5%)	2874 (58.5%)	<.0001	464 (55.7%)	2441 (56.6%)	.61	1711 (53.7%)	1179 (61.0%)	<.0001
Percutaneous coronary intervention, n (%)	2.464 (47.9%)	118 (51.5%)	2346 (47.8%)	.26	397 (47.7%)	2067 (48.0%)	.87	1645 (51.7%)	814 (42.1%)	<.0001
Coronary artery bypass grafting, n (%)	1.667 (32.4%)	82 (35.8%)	1585 (32.3%)	.26	254 (30.5%)	1413 (32.8%)	.19	1001 (31.4%)	659 (34.1%)	.15
Stroke or TIA, n (%)	404 (7.9%)	0	404 (8.2%)	<.0001	103 (12.4%)	301 (7.0%)	<.0001	165 (5.2%)	234 (12.1%)	<.0001
Carotid disease, n (%)	721 (14.0%)	52 (22.7%)	669 (13.6%)	<.0001	183 (22.0%)	538 (12.5%)	<.0001	295 (9.3%)	419 (21.7%)	<.0001
Congestive heart failure, n (%)	912 (17.7%)	4 (1.7%)	908 (18.5%)	<.0001	88 (10.6%)	824 (19.1%)	<.0001	215 (6.8%)	683 (35.3%)	<.0001
Hospitalization for hear failure, n (%)	200 (3.9%)	0	200 (4.1%)	<.0001	12 (1.7%)	186 (4.3%)	.0003	44 (1.4%)	149 (7.7%)	<.0001
Creatinine (μmol/L), mean ± SD	94 (26.2)	91.1 ± 19.8	94.1 ± 26.4	.09	91.8 ± 24.5	94.4 ± 26.5	.01	92.3 ± 24.6	96.6 ± 28.5	<.0001
Hypertension, n (%)	3945 (76.5%)	29 (12.7%)	3916 (79.7%)	<.0001	302 (36.3%)	3643 (84.5%)	<.0001	2447 (76.9%)	1484 (76.7%)	.10
Diabetes, n (%)	1753 (34.1%)	12 (5.2%)	1741 (35.4%)	<.0001	218 (26.2%)	1535 (35.6%)	<.0001	831 (26.1%)	911 (47.1%)	<.0001
Dyslipidemia, n (%)	4334 (84.3%)	177 (77.3%)	4157 (84.6%)	.03	746 (89.6%)	3588 (83.3%)	<.0001	2677 (84.1%)	1637 (84.6%)	.75
Actual smoker, n (%)	613 (11.9%)	39 (17.0%)	574 (11.7%)	.008	96 (11.5%)	517 (12.0%)	.0005	248 (7.8%)	361 (18.7%)	<.0001
Risk score										
REACH Bleeding Risk Score, mean ± SD	8.0 ± 1.5	5.7 ± 1.3	8.1 ± 1.5	<.0001	5.4 ± 0.9	8.5 ± 1.1	<.0001	7.6 ± 1.5	8.6 ± 1.3	<.0001
REACH Recurrent Ischemic Score, mean ± SD	12.0 ± 2.5	10.6 ± 2.0	12.1 ± 2.5	<.0001	10.7 ± 2.0	12.2 ± 2.5	<.0001	10.4 ± 1.3	14.5 ± 1.5	<.0001
CHA_2_DS_2_VaSc Score, mean ± SD	3.3 ± 1.2	1 ± 0.2	3.4 ± 1.1	<.0001	2.4 ± 1.1	3.5 ± 1.1	<.0001	3.1 ± 1.1	3.7 ± 1.2	<.0001
**Medications**										
Aspirin, n (%)	4682 (91.1%)	199 (86.9%)	4483 (91.2%)	.02	755 (90.6%)	3927 (91.1%)	.64	2960 (93.0%)	1703 (88.1%)	<.0001
Other antiplatelet therapy, n (%)	342 (6.7%)	25 (10.9%)	317 (6.5%)	.008	38 (4.6%)	304 (7.1%)	.008	188 (5.9%)	152 (7.9%)	.02
Statins, n (%)	4352 (84.7%)	189 (82.5%)	4165 (84.8%)	.36	715 (85.8%)	3639 (84.5%)	.31	2904 (91.2%)	1443 (74.6%)	<.0001
Beta‐blockers, n (%)	3895 (75.7%)	149 (65.1%)	3747 (76.2%)	.001	590 (70.8%)	3305 (76.7%)	.0003	2375 (74.6%)	1503 (77.7%)	.01
ACE inhibitors or ARBs, n (%)	3992 (77.6%)	87 (38.0%)	3905 (79.5%)	<.0001	525 (63.0%)	3467 (80.5%)	<.0001	2420 (76.0%)	1554 (80.4%)	.001

Abbreviations: ACE, angiotensin‐converting enzyme; ARB, angiotensin II receptor blocker; BMI, body mass index; TIA, transient ischemic attack.

### 3.2 Distribution of the three risk score in the COMPASS‐eligible population

In the overall COMPASS‐eligible population, mean CHA_2_DS_2_VaSc score, REACH RIS and REACH BRS were 3.3 ± 1.2, 12.0 ± 2.5, and 8.0 ± 1.5, respectively **(**Table 1 **)**. Only 4.5% (n = 229) of the population was categorized as low CHA_2_DS_2_VaSc, while the vast majority of patients (95.5%, n = 4.913) were in the high CHA_2_DS_2_VaSc category. Regarding the REACH BRS score, 16.2% (n = 833) of the patients were categorized in the “low” group and 83.8% (n = 4.309) in the “intermediate” group. Finally, 61.9% (n = 3.184) of the COMPASS‐CLARIFY patients were in the “low” REACH RIS group, and 37.6% (n = 3.184) in the intermediate REACH RIS. Very few patients (0.3%, n = 18) had a high REACH RIS, and thus will not be considered in the analysis. **(**Table 2 **).**

**TABLE 2:** Distribution of risk score categories in the COMPASS‐eligible population within the CLARIFY registry

	REACH Recurent Ischemic Score	REACH Bleeding Risk Score	CHA_2_DS_2_VaSc Score	
Low (n, %)	3184 (61.9%)	833 (16.2%)	229 (4.4%)
Intermediate (n, %)	1934 (37.6%)	4309 (83.8%)		
High (n, %)	18 (0.3%)	Excluded	4913 (95.5%)	

*Note*: The REACH recurent ischemic score ranges as follows: low from 0 (< 1% of risk) to 12 (5.4% of risk), intermediate from 13 (6.3% of risk) to 19 (15.0% of risk) and high for patients above ≥20 (> 17% of risk over 20 months). The REACH bleeding risk score ranges as follows: low from 0 to 6 (0.46% of risk over a two‐year period), intermediate between 7 and 10 (approximately 1.1% of risk) and high above 10 (> 2.76% of risk). Low‐intermediate CHA_2_DS_2_VaSc score ranges between 0 and 1, and high CHA_2_DS_2_VaSc is for patients ≥2.

### 3.3 Ischemic outcomes according to risk scores

**FIGURE 1:**
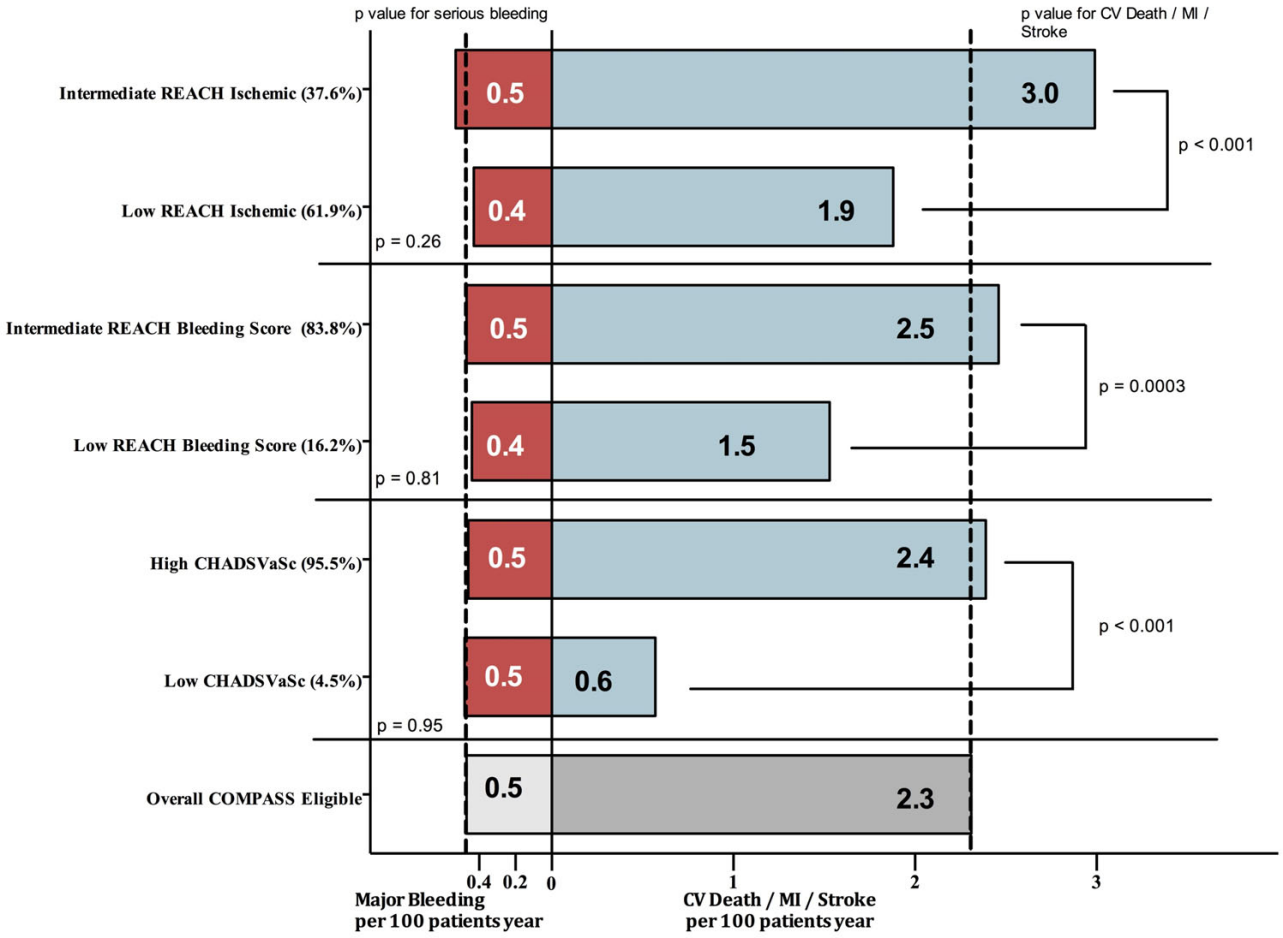
Ischemic and bleeding outcomes in the COMPASS‐eligible population subset of the CLARIFY registry according to the REACH RIS, REACH BRS and CHADSVASC score. This figure depicts both ischemic outcomes on the right side, for the overall COMPASS‐CLARIFY‐eligible population (in dark gray) and according to the three risk scores (in blue), and bleeding on the left side, for the overall COMPASS‐CLARIFY‐eligible population (in light gray) and according to the three risk scores (in red). Compared to the overall population, patients with intermediate REACH RIS have a markedly increase in ischemic outcome, without increased bleeding risk. Patients with low CHA_2_DS_2_VaSc have a remarkably low ischemic risk, and similar bleeding risk. All risk scores demonstrate poor performance for identifying patients at higher risk of bleeding. REACH BRS, REACH Bleeding Risk Score; REACH RIS, REACH Recurrent Ischemic Score

The rate of the composite outcome of CV death, MI or stroke in the overall COMPASS‐eligible population was 2.3 per 100 patients years (95% CI 2.1–2.5). Compared to the overall population, patients with intermediate REACH RIS had the highest ischemic risk (3.0 [2.6–3.4]) followed by intermediate REACH BRS (2.5 [2.2–2.7]) and high CHA_2_DS_2_VaSc (2.4 [2.2–2.6]) (Figure 1).

Additionally, patients categorized in the lowest range of each of the three scores, had a lower ischemic risk than the overall COMPASS‐CLARIFY‐eligible population, especially patients in the low‐ CHA_2_DS_2_VaSc group (1.9 [1.7–2.1] for REACH RIS, 1.5 (1.2–2.0])for REACH BRS, and 0.6 (0.3–1.3) for CHA_2_DS_2_VaSc).

Intermediate REACH RIS or REACH BRS, and high CHA_2_DS_2_VaSc were associated with a significantly higher risk of occurrence of the composite ischemic outcome, compared to low REACH RIS, REACH BRS or CHA_2_DS_2_VaSc, respectively (3.0 [2.6–3.4]) versus 1.9 (1.7–2.1), *p* < .001; 2.5 (2.2–2.7) versus 1.5 (1.2–2.0), *p* < .001; and 2.4 (2.2–2.6) versus 0.6 (0.3–1.3), *p* < .001.

### 3.4 Bleeding according to risk scores

The rate of bleeding in the overall COMPASS‐eligible population was 0.5 per 100 patient years (95% CI, 0.4–0.6). There were no differences between the rates of bleeding in patients with intermediate versus low REACH RIS (0.5 [95% CI, 0.4–0.7] vs. 0.4 [95% CI, 0.3–0.5] per 100 patient years, *p* = .26), intermediate versus low REACH BRS (0.5 [95% CI, 0.4–0.6] vs. 0.4 [95% CI, 0.3–0.7], *p* = .81), and high vs low CHA_2_DS_2_VaSc score (0.5 [95% CI, 0.4–0.6] vs. 0.5 [95% CI, 0.2–1.1], *p* = .95), as well as between any of these risk categories and the overall study population (Figure 1).

## 4 DISCUSSION

In the present analysis, we sought to evaluate the performance of three risk scores to identify within COMPASS‐Eligible patients from the CLARIFY registry, those with the most favorable trade‐off between ischemic and bleeding events. The main results of our analysis are that a low CHA_2_DS_2_VaSc score (0 or1) identifies, admittedly a small proportion of patients, but at very low ischemic risk and at similar bleeding risk. Thus, these patients appear unlikely to benefit from a combination of low‐dose rivaroxaban and aspirin. On the other hand, patients with an intermediate REACH RIS had a markedly higher ischemic risk, but with no apparent increased risk of bleeding, and thus appear to be the optimal candidates for the COMPASS regimen. Finally, all three risk scores used in this study demonstrated poor performance in identifying patients at higher or lower bleeding risk, and probably should not be used for evaluation of the risk of bleeding in COMPASS‐eligible patients.

Bleeding risk remains a major concern when using antithrombotic therapies in clinical practice, with the fear of causing harm with an overt bleed while the ischemic events prevented are clinically “silent” and thus unrecognized, leading to a distorted perception of benefits and harms of treatment. There have been several attempts to identify patients with an optimal benefit–risk ratio for the prescription of low dose rivaroxaban and aspirin using simple clinical criteria such as diabetes, heart failure, number of diseased vascular beds or renal failure.^19^, ^20^ Additionally, we^21^ and others^20^ have found that the combination of multiple enrichment criteria resulted in a dramatic increase of ischemic risk without significant increase in bleeding risk suggesting that these patients may derive the greatest benefit from the COMPASS regimen. However, to date, the performance of risk scores has never been evaluated for this purpose.

This score‐based approach for clinical management and risk stratification of patient has already proven to be effective in other clinical settings, such as the use of the GRACE score^22^ for risk stratification in acute coronary syndromes, or the PRECISE‐DAPT,^23^ DAPT^24^ or the ABC‐CHD^25^ scores in decision making regarding duration of dual antiplatelet therapy after PCI, and is recommended by guidelines.^8^ The rationale for evaluating both the REACH RIS^16^ and BRS^17^ is that these two scores have been developed and externally validated in a large registry of more than 68 000 outpatients with chronic vascular disease,^18^ somewhat similar to the COMPASS Trial population. The CHA_2_DS_2_VaSc score has been validated for stroke prediction in patients with AF but based on the individual components of this score and its ease of use in clinical practice, several studies have also demonstrated its value for predicting ischemic events or mortality in various settings, independent of the presence of AF.^26^, ^27^, ^28^, ^29^, ^30^

If these three scores performed well in identifying patients at high or low risk of ischemic events in the present study, they did not allow accurate evaluation of the risk of bleeding. Such a discrepancy was unexpected, in particular for the REACH BRS but may be explained by the fact that serious bleeding events were relatively rare in the study population (108 events, 0.5 per 100 patients years), and that the COMPASS‐eligible population accounted for approximately one out of three patients with CCS, and around half with chronic vascular disease,^31^ with higher risk patients being excluded.

Interestingly, several items used in the calculation of these scores are redundant, and their performance only depends on the weighting of each item. The REACH RIS involves more items with a larger score range (from 0 to >29) and may be slightly more difficult to compute, but this may explain its better distribution in the study population and its enhanced performance for prediction of ischemic events.

Several limitations of this study must be acknowledged. First, the definition of “serious bleeding” did not include fatal bleeding, as in the modified ISTH definition used in COMPASS. However, and apart from fatal bleeding, other bleeding defined as “major” by the COMPASS protocol were captured using our definition. Additionally, this definition of bleeding used for developing the REACH BRS was externally validated in the CHARISMA trial. Second, we analyzed the ischemic and bleeding outcomes within a population of CCS patients, currently under single antiplatelet therapy. There remains uncertainty whether the ischemic and bleeding risk will evolve once patients would be under combination of aspirin and rivaroxaban.. Finally, approximately one half of the overall CLARIFY population was excluded from the analysis due to missing data regarding evaluation of eligibility to COMPASS, which may affect the applicability of the results.

## 5 CONCLUSIONS

Within a broad population of patients with chronic coronary syndromes fulfilling the COMPASS eligibility criteria, a low CHA_2_DS_2_VaSc score identify a relatively small subset of patients unlikely to benefit from combination of low‐dose rivaroxaban and aspirin, while patients with an intermediate REACH Recurent Ischemic Score appear as attractive candidates for this strategy. All scores demonstrated poor performance for the prediction of bleeding events in this population.

### ACKNOWLEGMENTS

The CLARIFY registry was supported by Servier. The sponsor had no role in initiating the present analysis, nor in the study design, data analysis and interpretation or in the decision to submit the manuscript for publication.

## CONFLICT OF INTEREST

Dr Arthur Darmon discloses the following: research grants from Abbott and travel fees by AlviMedica and Bayer. Professor Gregory Ducrocq discloses speaker's and/or consulting fees from Amgen, Astra Zeneca, Bayer, BMS, Janssen, Sanofi, Terumo. Adam Jasilek has nothing to disclose. Professor Laurent Feldman has nothing to disclose. Dr Emmanuel Sorbets reports personal fees and non‐financial support from Servier, during the conduct of the study; personal fees and non‐financial support from Novartis, personal fees and non‐financial support from Bayer, personal fees and non‐financial support from Astra‐Zeneca, personal fees and nonfinancial support from Merck Sharpe & Dohme, personal fees from Bristol‐Myers Squibb, outside the submitted work. Dr Roberto Ferrari reports grants and personal fees from Servier International and Novartis, and personal fees from Merck Serono, Bayer, and Boehringer Ingelheim outside the submitted work. Dr Ian Ford reports research grants and personal fees from Servier in relation to the CLARIFY registry. Pr Jean‐Claude Tardif reports research grants from Amarin, Astra‐Zeneca, DalCor, Esperion, Ionis, RegenXBio, Sanofi and Servier; honoraria from DalCor, HLS Therapeutics, Sanofi and Servier; holds minor equity interest in DalCor; and is an author of a patent on pharmacogenomics‐guided CETP inhibition. Pr Michal Tendera declares honoraria from Servier for participation to the Executive Committee of the CLARIFY registry, related to the submitted work, and the following activities outside the submitted work: consultancy and speakers fees from Bayer and Cadila Pharmaceuticals; consultancy fees from Janssen‐Cilag, Kowa, OncoArendi, PERFUSE Group, Servier and UCB Pharmaceuticals. Dr. Kim M. Fox reports personal fees and other from Servier, during the conduct of the study; personal fees and other from Celixir, personal fees and other from UCH, personal fees and other from Broadview Ventures, other from AstraZeneca, outside the submitted work; and Director of Vesalius Trials. Pr Philippe Gabriel Steg declares the following: research grants from Amarin, Bayer, Sanofi, and Servier, Clinical trials (Steering committee or CEC or DMC), speaker or consultant: Amarin, AstraZeneca, Bayer, Boehringer Ingelheim, Bristol‐Myers Squibb, Idorsia, Mylan, Novo‐Nordisk, Novartis, Pfizer, Sanofi, Servier.

## Data Availability

The data that support the findings of this study are available on request from the corresponding author. The data are not publicly available due to privacy or ethical restrictions.
